# Household energy efficiency and health: Area-level analysis of hospital admissions in England

**DOI:** 10.1016/j.envint.2019.105164

**Published:** 2019-12

**Authors:** R.A. Sharpe, K.E. Machray, L.E. Fleming, T. Taylor, W. Henley, T. Chenore, I. Hutchcroft, J. Taylor, C. Heaviside, B.W. Wheeler

**Affiliations:** a European Centre for Environment and Human Health, University of Exeter Medical School, Knowledge Spa, Royal Cornwall Hospital, Truro, Cornwall TR1 3HD, United Kingdom; b Public Health, Cornwall Council, 1E, New County Hall, Truro TR1 3AY, United Kingdom; c Health Statistics Research Group, Institute of Health Research, University of Exeter Medical School, St Luke’s Campus, Exeter, EX1 2LU, United Kingdom; d NHS NEW Devon Clinical Commissioning Group, County Hall, Exeter EX2 4QD, United Kingdom; e Regen, Bradninch Court, Castle Street, Exeter EX4 3PL and Energiesprong UK Limited, National Energy Centre, Davy Avenue, Knowlhill, Milton Keynes MK5 8NG, United Kingdom; f UCL Institute for Environmental Design and Engineering, UCL, 14 Upper Woburn Plc, London WC1H 0NN, United Kingdom; g Environmental Change Institute, University of Oxford, South Parks Road, Oxford OX1 3QY, Oxford, United Kingdom

**Keywords:** CCG, Clinical Commissioning Group, COA, Census Output Areas, COPD, chronic obstructive pulmonary disease, CVD, cardiovascular disease, EPC, energy performance certificate, EST, Energy Saving Trust, HA, Home Analytics, HEED, Household Energy Efficiency Database, IMD, Index of Multiple Deprivation, LSOA, Lower-layer Super Output Areas, MEDMI, The Medical and Environmental Data Mash-up Infrastructure, NHS, National Health Service, ONS, Office of National Statistics, SAP, Standard Assessment Procedure, Household energy efficiency, Fuel poverty, COPD, Asthma and cardiovascular disease

## Abstract

**Introduction:**

Fuel poverty affects up to 35% of European homes, which represents a significant burden on society and healthcare systems. Draught proofing homes to prevent heat loss, improved glazing, insulation and heating (energy efficiency measures) can make more homes more affordable to heat. This has prompted significant investment in energy efficiency upgrades for around 40% of UK households to reduce the impact of fuel poverty. Despite some inconsistent evidence, household energy efficiency interventions can improve cardiovascular and respiratory health outcomes. However, the health benefits of these interventions have not been fully explored; this is the focus of this study.

**Methods:**

In this cross sectional ecological study, we conducted two sets of analyses at different spatial resolution to explore population data on housing energy efficiency measures and hospital admissions at the area-level (counts grouped over a 3-year period). Housing data were obtained from three data sets covering housing across England (Household Energy Efficiency Database), Energy Performance Certificate (EPC) and, in the South West of England, the Devon Home Analytics Portal. These databases provided data aggregated to Lower Area Super Output Area and postcode level (Home Analytics Portal only). These datasets provided measures of both state (e.g. EPC ratings) and intervention (e.g. number of boiler replacements), aggregated spatially and temporally to enable cross-sectional analyses with health outcome data. Hospital admissions for adult (over 18 years) asthma, chronic obstructive pulmonary disease (COPD) and cardiovascular disease (CVD) were obtained from the Hospital Episode Statistics database for the national (1st April 2011 to 31st March 2014) and Devon, South West of England (1st April 2014 to 31st March 2017) analyses. Descriptive statistics and regression models were used to describe the associations between small area household energy efficiency measures and hospital admissions. Three main analyses were undertaken to investigate the relationships between; 1) household energy efficiency improvements (i.e. improved glazing, insulation and boiler upgrades); 2) higher levels of energy efficiency ratings (measured by Energy Performance Certificate ratings); 3) energy efficiency improvements and ratings (i.e. physical improvements and rating assessed by the Standard Assessment Procedure) and hospital admissions.

**Results:**

In the national analyses, household energy performance certificate ratings ranged from 37 to 83 (mean 61.98; Standard Deviation 5.24). There were a total of 312,837 emergency admissions for asthma, 587,770 for COPD and 839,416 for CVD. While analyses for individual energy efficiency metrics (i.e. boiler upgrades, draught proofing, glazing, loft and wall insulation) were mixed; a unit increase in mean energy performance rating was associated with increases of around 0.5% in asthma and CVD admissions, and 1% higher COPD admission rates. Admission rates were also influenced by the type of dwelling, tenure status (e.g. home owner versus renting), living in a rural area, and minimum winter temperature.

**Discussion:**

Despite a range of limitations and some mixed and contrasting findings across the national and local analyses, there was some evidence that areas with more energy efficiency improvements resulted in higher admission rates for respiratory and cardiovascular diseases. This builds on existing evidence highlighting the complex relationships between health and housing. While energy efficiency measures can improve health outcomes (especially when targeting those with chronic respiratory illness), reduced household ventilation rates can impact indoor air quality for example and increase the risk of diseases such as asthma. Alternatively, these findings could be due to the ecological study design, reverse causality, or the non-detection of more vulnerable subpopulations, as well as the targeting of areas with poor housing stock, low income households, and the lack of “whole house approaches” when retrofitting the existing housing stock.

**Conclusion:**

To be sustainable, household energy efficiency policies and resulting interventions must account for whole house approaches (i.e. consideration of the whole house and occupant lifestyles). These must consider more alternative ‘greener’ and more sustainable measures, which are capable of accounting for variable lifestyles, as well as the need for adequate heating and ventilation. Larger natural experiments and more complex modelling are needed to further investigate the impact of ongoing dramatic changes in the housing stock and health.

**Study implications:**

This study supports the need for more holistic approaches to delivering healthier indoor environments, which must consider a dynamic and complex system with multiple interactions between a range of interrelated factors. These need to consider the drivers and pressures (e.g. quality of the built environment and resident behaviours) resulting in environmental exposures and adverse health outcomes.

## Highlights

• People living in areas with higher energy efficiency levels had increased risk of hospitalisation for asthma, COPD and CVD.
• Admission rates were also associated with location/climate, the type of dwelling, and tenure status.
• Findings suggest there may be need for whole house approaches when retrofitting existing housing stock.

## 1 Introduction

Mitigating and adapting to climate change represents a major worldwide challenge (D'Amato et al., 2015). Failure to respond to the challenge of climate change will have diverse health and wellbeing consequences, as well as social and political ramifications (McMichael, 2014; ONS, 2014; Smith et al., 2014). In the UK, housing represents around 25% of total UK CO_2_ emissions (Hamilton et al., 2015) and represents a considerable public health concern. For example, in the UK, cold related mortality is about 20 times higher than heat related mortality, and will continue to be a cause for concern in future decades, partly due to an increasing and ageing UK population (Hajat et al., 2014). This has prompted significant investment in energy efficiency upgrades for around 40% of UK households to provide the “co-benefits” of reducing the domestic carbon footprint and fuel poverty alleviation (Dear and McMichael, 2011; Liddell and Morris, 2010; Thomson et al., 2013). Households living in fuel poverty are a major public health priority because fuel poverty affects up to 34% of European homes (Liddell and Morris, 2010; Thomson et al., 2013). In the UK, it has been estimated that a fifth of excess winter deaths are attributable to the coldest quarter of homes (Public Health England, 2014). Living in cold homes is associated with a range of physical and mental health effects; and is known as a risk factor for cardiovascular and respiratory diseases (Barnett et al., 2005; Public Health England, 2014; Sharpe et al., 2015b; Shiue and Shiue, 2014).

While the definition of fuel poverty varies (Liddell et al., 2012; Middlemiss, 2016; Moore, 2015), it is driven by interactions between household income, the current cost of energy, the energy efficiency level of the home and resident behaviours (PHE, 2014). Those most vulnerable to living in cold homes include low income households, the very young, elderly/infirm, and hard to reach populations such as those living in the private rental sector (Sharpe et al., 2015c; Sharpe et al., 2015d). While lower income households are also more likely to live in deprived neighbourhoods with poor and inefficient housing (further impacts on the ability to adequately heat and ventilate the home) (Committee on Fuel Poverty, 2016), they are also more likely to be supported by fuel poverty policy interventions and receive funding for energy efficiency measures. This increases the number of lower income households receiving fabric interventions (e.g. insulation and new heating systems) when assessed at the area-level (Hamilton et al., 2014). The impact of household income is important because those living in fuel poverty have to make stark choices about heating and ventilating their home by reducing energy use and fuel consumption (Anderson et al., 2012; Critchley, 2007).

Excess cold is considered a category 1 hazard (i.e. poses a serious and immediate risk to a person's health and safety) by the UK Housing Health and Safety Rating System (UK Office of the Deputy Prime Minister, 2006). Delivering remediation actions has the potential to significantly improve public health outcomes. In the UK, it has been estimated that the implementation of fuel poverty interventions could save the UK National Health Service (NHS) more than £800 m per year (Nicol et al., 2015). This includes household energy efficiency improvements such as sealing homes to prevent heat loss (e.g. draughtt proofing, glazing and insulation) and improved heating systems (Sharpe et al., 2015d). These improvements have the potential to reduce the risk of cold-related illnesses by making homes more affordable to heat (Hodges et al., 2016; Maidment et al., 2014; Powell et al., 2017; Thomson et al., 2013).

Energy efficiency interventions are continuing without considering the long-term impacts on health (Bone et al., 2010). Previous interventions have resulted in a small but significant improvement on health (Maidment et al., 2014). However the evidence is inconsistent and interventions may have a smaller effect on certain health outcomes such as respiratory diseases (Bone et al., 2010; Thomson et al., 2013). A larger scale natural experimental study of the Warm Homes Nest Scheme evaluated health outcomes during the winter before and winter following the intervention. The study indicated a positive effect of intervention on respiratory outcomes, and suggested evidence of reduced emergency hospital admissions for cardiovascular and respiratory conditions (Welsh Government, 2017).

While this relatively short term follow up can reasonably infer causality for the immediate impact of the intervention, it does not consider the long term impact of energy efficiency improvements on health. This is important to consider because of the potential unintended consequences of reduced ventilation rates from sealing homes to prevent heat loss and the subsequent impact on indoor air quality (Shrubsole et al., 2014). Previous interventions have resulted in short-term improvements in the indoor environment (Richardson et al., 2005), but are also thought to increase the risk of asthma (Sharpe et al., 2015d). These can result from reduced household ventilation rates and changes in indoor temperatures, relative humidity, and air quality (Shrubsole et al., 2014). While household energy efficiency measures can achieve small improvements on health, these factors may explain some of the inconsistent findings of some prior interventions (Maidment et al., 2014).

While increased energy efficiency makes homes more affordable to heat, these improvements do not eliminate the impact of cold on the lowest income households (Anderson et al., 2012), or account for variations in resident behaviours when heating or ventilating the home (Critchley, 2007). This is important to consider because fuel poor households are likely to suffer the impacts of damp and cold regardless of the perception of the potential health risks, use of ventilation, and the energy efficiency levels (Sharpe et al., 2015c). Furthermore, understanding the impact of energy efficiency interventions on health is compounded by prior studies with small sample sizes (Maidment et al., 2014; Thomson et al., 2001), and the lack of linkages made between built environment and health data at the population level (Sharpe et al., 2015d). To our knowledge, no study has assessed the relationship between energy efficiency interventions and health at the population-level.

In this study, we assessed whether area-level energy home energy efficiency ratings and improvements across England and Devon in the South West of England were associated with the risk of hospital admissions for cardiovascular and respiratory diseases (i.e. counts of hospital admission between April 2011 and March 2014, and between April 2014 and March 2017, respectively).

## 2 Methodology

This cross sectional ecological study involved two analysis streams with similar approaches, linking small-area, whole population data on housing energy efficiency and hospital admissions. The first analyses used data at Lower-layer Super Output Area (LSOA) level for England; the second used higher resolution postcode level data for one area of south west England (Devon). Each part of the study was designed to make best use of available energy efficiency and hospital data as previously described (Sharpe et al., 2018a) (Table 1).

**Table 1:** Data sets used in data analyses (Sharpe et al., 2018a).

Type of data	National analyses	Local analyses
Housing characteristics	The Home Energy Efficiency Database (HEED, Energy Saving Trust) provided data on household energy efficiency measures completed between 2007 and 2014.	The Devon Home Analytics database (Energy Saving Trust) provided data on loft insulation, glazing, boiler replacements, SAP (Standard Assessment Procedure) Ratings and the probability of fuel poverty.
Energy efficiency	Data from Energy Performance Certificates (EPCs) provided EPC ratings as a measure of energy efficiency.	N/A
Health	Hospital Episode Statistics (HES) were used to derive counts of admissions per LSOA, by sex and age group for a 3-year period (1st April 2011 to 31st March 2014). Admissions were selected for adults (aged 18+) admitted with asthma (ICD-10 codes J45 & J46), emphysema & chronic bronchitis, Chronic Obstructive Pulmonary Disease (COPD, ICD-10 J40-J44) and cardiovascular Disease (CVD), comprising hypertensive heart disease (I11), acute stroke (I60–69) & ischemic heart disease (excluding chronic) (I20–24)	Hospital Episode Statistics were made available by the NHS Northern, Eastern and Western Devon Clinical Commissioning Group (CCG). Emergency admissions of adults aged 18+ for asthma, COPD and CVD were extracted for three years, 1st April 2014 to 31st March 2017. Population denominators were obtained from the 2011 Census.
Covariates	• Indices of deprivation for 2010 were obtained from DCLG (Department for Communities and Local Government, 2011). • Urban-rural category for each LSOA was based on 2001 Census data (Office for National Statistics, 2004). • Modelled ambient air pollution data (Mukhopadhyay and Sahu, 2017) were obtained via the MEDMI project^a^ (Fleming et al., 2014). Annual averages of background concentrations of NO_2_, PM_2.5_, and ozone were obtained for a 1 km grid for the years 2007–11. • Data on long-term average weather parameters for the ten-year period 2006 to 2015 were obtained from the Met Office, also via the MEDMI platform.	• Income, employment and education deprivation scores for 2011 were obtained from the indices of deprivation for 2015 (Department for Communities and Local Government, 2015). • Urban-rural category (2011 Census) for each postcode was also extracted from the ONS Postcode Directory. Tenure and dwelling type distributions were aggregated to postcode level from the Home Analytics database. • MEDMI was used to provide air pollution and weather data.

a https://www.data-mashup.org.uk.

**Fig. 1:**
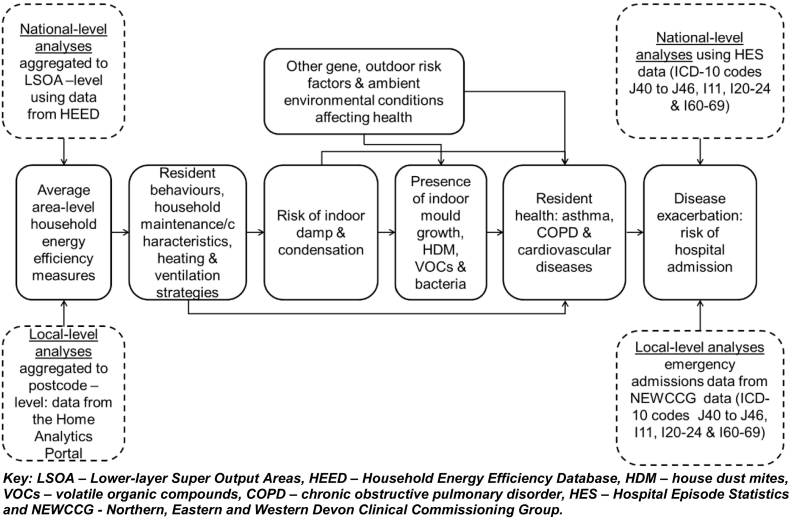
Concept model of energy efficiency & determinants of health. Key: LSOA – Lower-layer Super Output Areas, HEED – Household Energy Efficiency Database, HDM – house dust mites, VOCs – volatile organic compounds, COPD – chronic obstructive pulmonary disorder, HES – Hospital Episode Statistics and NEWCCG - Northern, Eastern and Western Devon Clinical Commissioning Group.

Adapted from Sharpe et al. (2015e) and Sharpe et al. (2015d).

Both analyses investigated the association between area-level summaries of dwelling energy efficiency improvements (e.g. boiler upgrades, improved insulation and glazing), as well as energy efficiency ratings (as a measure of the energy performance of a dwelling) and the risk of hospital admissions for asthma, chronic obstructive pulmonary disease (COPD) and cardiovascular disease (CVD). Fig. 1 outlines the conceptual framework underpinning the study, hypothesising the relationship between household energy efficiency levels, indoor exposures, and risk of health effects (adapted from Sharpe et al. (2015e) and Sharpe et al. (2015d)).

The first stream of analyses involved national (England) household data from the Household Energy Efficiency Database (HEED) and hospital admission data from Hospital Episodes Statistics (HES). To explore whether these findings were replicated at higher spatial resolution we used a novel household-level data set, the Devon Home Analytics Portal (available for the county of Devon), for a second analysis. In both analyses, we assessed associations between area average energy efficiency levels and hospital admission rates. Ethical approval was obtained from the University of Exeter Medical School Research Ethics Committee (ref FEB17/D/119).

### 2.1 National energy efficiency data

The Energy Saving Trust's Household Energy Efficiency Database (HEED) (Hamilton et al., 2014; Hamilton et al., 2013) provides a unique record of energy efficiency installations that have been implemented across the UK. Records of property attributes (build form, tenure, age, and fuel type) and installation data pertaining to heating systems, insulation, glazing, and draught proofing have been collected since 1992. While the counts of energy efficiency measures are unique (e.g. counts of draught proofing), there is the potential for multiple interventions per dwelling; this can skew the analyses when aggregating HEED records.

Duplicates were removed using data stamps associated with each record to prioritise the data in HEED; and, in any case where a property had multiple records for the same property attribute, the outdated records were removed. Once this de-duplication was performed at the address-level, the remaining records were then aggregated to LSOAs. These small areas, designed for the reporting of population statistics, have an average population of around 1600. Aggregated data for the following measures were derived from HEED for all LSOAs in Great Britain between the period from 2007 to 2014:

• Loft insulation – count of properties with a record of loft insulation >250 mm deep
• Wall insulation – count of properties that were built insulated or had a record of filled cavities, internal or external wall insulation
• Glazing type – count of properties with a record of fully double-glazed windows
• Draught proofing – count of properties with a record of draught proofing
• Boiler replacement – count of properties with at least one record of a boiler replacement
• Property age – count of properties built post-1995

Prior studies have used HEED data to describe the uptake rate or prevalence of household energy efficiency measures (Hamilton et al., 2014). While these data provide useful counts of installed measures, it is not possible to calculate rates (i.e. the percentage of properties in an area with a particular measure installed) because the database does not hold records for all properties. To calculate counts of HEED measures as a proportion of the housing stock, the HEED property counts for each LSOA were divided by the corresponding dwelling counts published by the Office of National Statistics (ONS) based on the 2011 census.

Energy Performance Certificates (EPCs) are another national source of dwelling energy efficiency data. EPCs are based on individual dwelling surveys conducted by professional assessors carried out any time a property is built, bought or sold, and provide an overall energy efficiency rating for the property. While they are not included in HEED, data from Energy Performance Certificates (EPCs) have recently been made available by The Ministry of Housing, Communities and Local Government (The Ministry of Housing, 2017). Data from the currently available EPCs (c. 12.4 million) were aggregated to LSOAs, to produce a mean and modal EPC rating for each area.

### 2.2 Devon home analytics database (postcode-level)

As described above, the HEED data are only available at the LSOA-level, which are still large enough to be relatively heterogeneous in terms of housing conditions. To overcome this limitation, we used household-level data available from the Energy Saving Trust Home Analytics (HA) Portal. This was commissioned in 2015 by Devon County Council, in conjunction with the unitary authorities of Plymouth and Torbay to estimate building attributes and household energy efficiency characteristics for all properties across Devon.

HA data were produced by Energy Saving Trust (EST) using data from household EPCs, historical installation records from HEED, council data, census information, and address details from the Ordnance Survey, linked using a Unique Property Reference Number (UPRN). These data were prioritised according to the most current and trusted information from these data sets (with EPCs being at the top of the hierarchy, followed by council data and HEED records) and grouped into more common classifications (e.g. according to the type of insulation). EPCs provided data on approximately 50% of the housing stock in Devon. In the Devon HA, property attributes and energy efficiency characteristics of the remaining housing stock were modelled and validated by EST using a set of geospatial algorithms. Data governance restrictions meant that individual patient-level data could not be linked at the household level and so, the analysis was conducted at postcode level (domestic, non-commercial postcodes include on average around 15 households). The HA property data for over 500,000 homes were therefore aggregated to postcodes:

• Loft insulation: The number of properties within each postcode with at least 250 mm deep loft insulation was calculated. Four categories of postcode were produced:
  o All properties in postcode have ≥250 mm deep loft insulation
  o None of the properties in the postcode have ≥250 mm deep loft insulation
  o The postcode contains properties with a mixture of loft insulation levels above and below 250 mm
  o All properties within the postcode had no loft (i.e. all flats)
• Wall insulation: in similar form to the loft insulation variable, three categories of postcode were produced:
  o All properties in postcode have insulated walls
  o None of the properties in the postcode have insulated walls
  o The postcode contains properties with a mixture of insulated and uninsulated walls
• Glazing: in similar form, postcodes were classified into 3 categories:
  o All properties in postcode double/triple glazed
  o All properties single glazed/partial double glazed
  o Postcode contains properties with a mixture of glazing
• SAP (Standard Assessment Procedure) Rating: The percentage of properties within each postcode with higher energy efficient homes with a corresponding SAP rating of A to C (ranges from A [highest] to G [lowest]).

Postcodes containing dwellings with a mixture of characteristics were used as the reference category in regression models, so that ‘good efficiency’ postcodes where all dwellings meet the efficiency criterion (e.g. loft insulation ≥250 mm deep) and ‘poor efficiency’ postcodes where none do were compared to ‘mixed’ postcodes. The SAP is the Government methodology for assessing and comparing the energy and environmental performance of dwellings; it is the chosen methodology for delivering the EU performance of the building directive, and used to calculate EPCs. Using SAP data allowed for comparison with other studies investigating the relationship between household energy efficiency and health (Kelly et al., 2012; Sharpe et al., 2015d).

### 2.3 Health outcomes

To link with the national and local data on property characteristics, hospital admission data relating to cold-related conditions for adults (aged 18+ years) were obtained. Emergency (i.e. unplanned) inpatient admissions relating to the following outcomes were included in both analyses:

• Asthma (ICD-10 codes J45 & J46)
• Emphysema & chronic bronchitis, Chronic Obstructive Pulmonary Disease (COPD, ICD-10 J40-J44)
• Cardiovascular Disease (CVD) comprising: hypertensive heart disease (I11), acute stroke (I60–69) & ischemic heart disease (excluding chronic) (I20–24)

Emergency admissions excluded those for planned procedures, but included patients arriving following immediate referral from primary care, or after attending the emergency department. In the national analyses, we used admissions data from the Hospital Episode Statistics (HES), which were obtained from the Health and Social Care Information Centre (now NHS Digital; see Acknowledgements). Counts of emergency admissions per LSOA, by sex and age group for a 3-year period (1st April 2011 to 31st March 2014) were derived from HES; and a subset of winter admissions (December–February each year) were also aggregated to enable focus on the key risk period for cold-related admissions. For the local analyses, hospital admission data comparable to HES were made available by the NHS Northern, Eastern and Western Devon Clinical Commissioning Group (NEW Devon CCG). Similarly, emergency admissions for asthma, COPD and CVD were extracted for three years, 1st April 2014 to 31st March 2017. Total 3-year counts of admissions per postcode, by sex and age group, were produced, along with 3-year winter admission totals.

HES counts ‘episodes’ separately (e.g. a new episode is generated when a patient is transferred); therefore, in both analyses only admission episodes were included and episodes generated by a transfer were excluded. Additionally, hospital admission data can include a large number of subsidiary diagnosis codes capturing comorbidities. In order to include only those admissions due primarily to the outcomes of interest, episodes were only included where the diagnosis code of interest appeared in the first or second diagnosis field.

To assess population rates of admissions at the LSOA and postcode-level, denominators from the 2011 Census were used. In national analyses, population denominators for each 2011 LSOA by age and sex were obtained and re-aggregated to 2001 boundaries using the GeoConvert platform (http://geoconvert.mimas.ac.uk), since admission counts were produced for 2001 Census LSOA boundaries. For the local analyses, 2011 postcode total populations were also available from the Census, but only disaggregated by sex. In order to generate estimated postcode populations by sex and age group for Devon, age/sex distributions were obtained for Census Output Areas (COA), within which postcodes nest, with a mean of around 10 postcodes per COA. Postcode populations were then distributed across age groups according to the demography of the containing COA.

### 2.4 Covariates

Complex interactions between a range of genetic and environmental factors influence the risk of the outcomes of interest (Fig. 1) (Sharpe et al., 2014). To account for this to the extent possible, we adjusted for a range of confounders/covariates in each analysis.

For the national analysis stream, urban-rural category for each LSOA was based on 2001 Census data (Office for National Statistics, 2004), classifying each LSOA/postcode as urban, town and fringe or rural. Urban-rural category (2011 Census) for each postcode was also extracted from the ONS Postcode Directory, again classifying each as urban, town and fringe, or rural. Census 2011 data on tenure (percentage of households renting from private or social housing providers) and dwelling types (percentage of household spaces that were flats) were obtained, and re-aggregated to 2001 LSOA boundaries. For the local analyses, tenure and dwelling type distributions were aggregated to postcode level from the Home Analytics database.

The Medical and Environmental Data Mash-up Infrastructure (MEDMI) project connected diverse climate, environment, and human health databases (Fleming et al., 2014). Ambient air pollution data from MEDMI were high resolution modelled estimates based on data from the UK air pollution monitoring network combined with a UK-specific air quality dispersion model, empirically verified through cross-validation (Mukhopadhyay and Sahu, 2017). Annual averages of background concentrations of NO_2_, PM_2.5_, and ozone were obtained for a 1 km grid (for the years 2007–11); and aggregated to the LSOA boundaries for national analyses and postcode point locations for local analyses. In addition, long-term average weather parameters (period 2006 to 2015) were obtained from the MEDMI platform. Monthly means of daily temperature; mean monthly precipitation (geometric mean); minimum winter temperature for the winter (December–February); and mean relative humidity were interpolated to a 5 km grid and aggregated to LSOA boundaries and postcodes using area-weighted interpolation.

To account for the impact of deprivation in the national analyses, the Indices of Deprivation for 2010 were obtained from the Ministry of Housing Communities and Local Government (2011). These data provided information about multiple aspects of deprivation for each LSOA. Seven domains make up a composite Index of Multiple Deprivation: income; employment; health and disability; education, skills and training; barriers to housing and services; living environment; and crime. For the purposes of this study, we used the income, employment and education deprivation scores as key measures of population socio-economic deprivation that could be related to both outcome and exposure measures. The three domain metrics are on different scales according to their source data and construction, but in each case, a higher score reflects a higher degree of deprivation in that domain for the LSOA population. Similarly, in the local analyses income, employment and education deprivation scores for 2011 LSOA boundaries were allocated to postcode point locations using the ONS Postcode Directory (ONS, 2015).

### 2.5 Data linkage

For the national study, energy efficiency, hospital admission and covariate datasets were all generated at LSOA-level using 2001 LSOA coding. These datasets could therefore be linked on a simple 1:1 basis. For the local analyses, in order to maintain confidentiality, the research team provided Northern, Eastern and Western (NEW) Devon Clinical Commissioning Group (CCG) with a postcode-level dataset of energy efficiency metrics and covariates. These were linked to hospital admissions data by CCG staff; and the full, linked dataset released to the research team with postcode identifiers removed. Although de-identified, for data governance purposes, the linked data could not be removed from the site and were only analysed on CCG computer systems.

### 2.6 Statistical analysis

The two linked datasets were explored through the production of basic descriptive statistics and simple bivariate analyses of total counts of hospital admissions across categories of key variables of interest. Following these initial explorations of the data, the core statistical analyses involved the development of sequential regression models testing hypotheses of the generic form: populations living in areas (LSOAs or postcodes) with higher average home energy efficiency would have lower admission rates for asthma, COPD or CVD than areas with lower average energy efficiency. Sub-hypotheses were tested to investigate: 1) whether the association would be stronger when analyses focused on winter months alone, and 2) whether the association would be stronger in areas with colder than average temperatures.

The form of the response variables (counts of admissions by area, age group and sex) suggested that Poisson-type regression models would be most appropriate, with inclusion of stratum-specific population as an offset. The large number of strata within each dataset containing zero admissions and exploration of descriptive statistics indicated the likely presence of over-dispersion in admission counts (variance > mean), violating the assumptions of Poisson regression models. Therefore, exploratory negative binomial models were conducted using the Stata ‘nbreg’ command (StataCorp, College Station). These models permit the comparison of Poisson and negative binomial models fit to the same data. The alpha test-statistic generated indicated in each case that over-dispersion was indeed present leading to negative binomial regression models being applied throughout. The Stata ‘vce(cluster)’ option was specified to allow for age group/sex strata observations clustered within LSOAs or postcodes.

For both national and local analyses, a common model building approach was adopted. In each case, a crude model was initially run, investigating the possible associations between admissions and energy efficiency variables adjusted for age and sex only. Subsequent models adjusted for potential confounders through the inclusion of the indices of deprivation, indicators of property and tenure types, urban/rural classification, air pollution, and weather variables.

Models were run for each of the three health outcomes (asthma, COPD, CVD) as 3-year total admission counts; and then with winter-only admissions to investigate the first sub-hypothesis. To explore the second sub-hypothesis, interaction tests were carried out to investigate the presence of effect modification between energy efficiency metrics and minimum temperature, applying likelihood ratio tests to compare models including and excluding interaction terms.

## 3 Results

Results are presented first for the national-level, and subsequently for the local-level analyses.

### 3.1 National-level

#### 3.1.1 Descriptive statistics

The total counts and rates of hospital admissions varied considerably by the health outcome of interest, by age, sex and season for the 3-year period: April 2011 to March 2014. Included in this dataset, there were a total of 312,837 emergency admissions for asthma, 587,770 for COPD, and 839,416 for CVD. The three winter months accounted for 27% of asthma, 30% of COPD, and 25% of CVD admissions. Men accounted for around 33% of hospital admissions for asthma, around 50% of the admissions for COPD, and 56% for CVD. For each of the admission count variables, the standard deviation was between 1.4 and 2.9 times the value of the mean, indicating the presence of over-dispersion, and justifying subsequent exploration using negative binomial models.

Table 2 presents descriptive statistics for energy efficiency metrics and covariates across the LSOAs. The extent and mixture of household energy efficiency improvements (loft/wall insulation, glazing, boiler replacement and boiler upgrades) varied considerably between areas. The mean EPC rating was 62.0 (Standard Deviation 5.2; range 37 to 83).

**Table 2:** Descriptive statistics for LSOA-level variables.

Variable	Mean	Std Dev	Min	Max
Home energy efficiency metrics^a^				
Loft insulation ≥250 mm deep per 100 houses	24.7	10.8	0.0	100.0
Wall insulation present per 100 houses/flats	2.7	2.8	0.0	72.0
Full double/triple glazing present per 100 houses/flats	6.6	4.5	0.0	69.5
Rate of draught proofing measures per 100 houses/flats	31.3	11.6	0.9	91.9
Rate of boiler replacement measures per 100 houses/flats	0.3	1.1	0.0	60.1
Mean EPC Rating	62.0	5.2	37.0	83.0

Indices of Deprivation 2010				
Income deprivation score	0.15	0.11	0.00	0.77
Employment deprivation score	0.10	0.07	0.00	0.75
Education deprivation score	21.7	18.8	0.0	99.3

Tenure/property types				
% Households private rented	16.0	11.4	1.3	87.9
% Households social rented	17.4	17.3	0.0	92.5
% Dwellings flats	20.0	22.2	0.0	99.7

Weather & air pollution				
Minimum winter temperature 2006–15 (C)	−4.4	0.8	−7.9	−1.0
Mean monthly precipitation 2006–15 (mm)	53.3	13.1	32.6	205.3
Mean relative humidity 2006–15 (%)	81.4	1.7	76.0	88.5
Mean NO2 2007–11 (ug/m^3^)	37.8	7.7	21.6	69.0
Mean ozone 2007–11 (ug/m^3^)	57.5	6.3	47.5	68.1
Mean PM_2.5_ 2007–11 (ug/m^3^)	12.8	0.5	11.0	17.7

Urban/rural classification (n)				
Urban	26,455			
Town & Fringe	3081			
Rural	2945			

a Measures per 100 dwellings indicate the rate of energy efficiency measures recorded in HEED 2007–2014 with 2011 Census counts as the denominator.

#### 3.1.2 Regression analyses

**Fig. 2:**
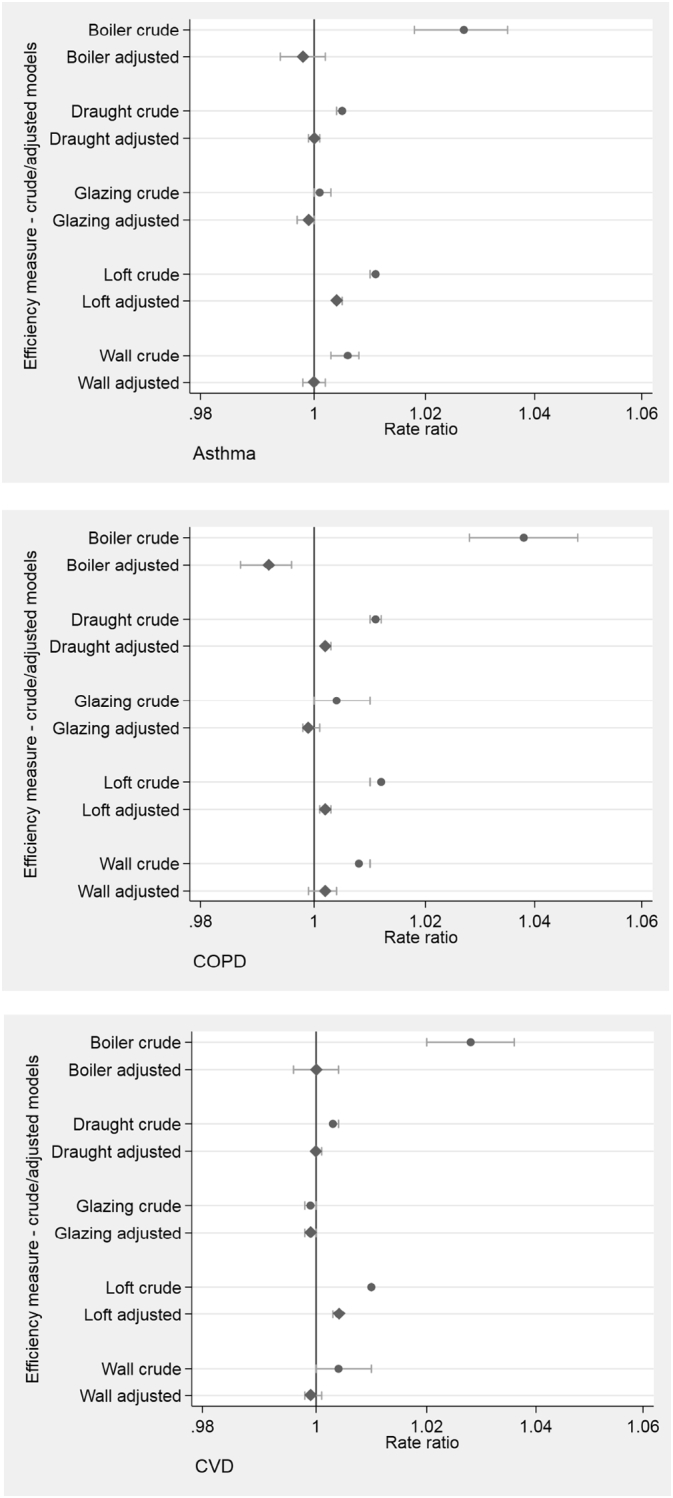
HEED energy efficiency metric associations with 3-year total hospital admission rates (England). Note: Rate ratios for a unit increase in: Loft: % dwellings with ≥250 mm deep loft insulation; Wall: % dwellings with wall insulation; Glazing: % dwellings with full double/triple glazing; Draught: draught proofing measures per 100 dwellings; Boiler: boiler replacement measures per 100 dwellings.

Fig. 2 depicts the rate ratios for hospital admissions associated with a unit increase in energy efficiency metrics at LSOA-level. Positive associations with asthma admission rates were observed in crude models for four of the five energy efficiency metrics (loft insulation, wall insulation, draught proofing and boiler replacement). However, the associations were completely attenuated in all but one model after adjusting for covariates. In the fully adjusted model, a percentage point increase in dwellings with 250 mm + deep loft insulation was associated with a 0.4% increase in asthma admissions.

Similar associations were observed between the energy efficiency metrics and COPD and CVD in the crude models. Loft insulation improvements were positively associated with a 0.2% and 0.4% significant increase in admission rates for COPD and CVD, respectively, after adjustment. However, the results also indicated that areas with higher levels of boiler replacement and glazing replacement had slightly lower admission rates for COPD and CVD, respectively. Again, similar patterns were observed between the energy efficiency metrics and 3-year hospital admission rates during the winter months. Slightly lower admission rates for asthma and COPD were associated with double or triple glazing and boiler replacements, respectively. Full model results are presented in Supplementary material Table S1.

**Fig. 3:**
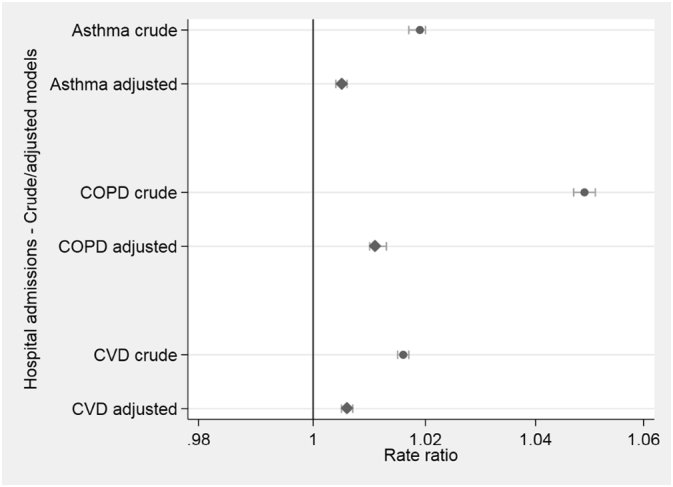
Energy Performance Certificate (EPC) Rating associations with 3-year total admission rates (England).

As described above, EPCs consider the overall energy efficiency rating of a dwelling. Crude models indicated associations between the higher LSOA mean EPC ratings and admission rates for asthma, COPD, and CVD. These associations persisted following adjustment for potential confounders, but were attenuated, with a unit increase in mean EPC rating associated with increases of around 0.5% in asthma and CVD admissions, and 1.0% higher COPD admission rates (see Fig. 3, and Supplementary Table S2).

In the full models, the associations between admissions and covariates (e.g. age, sex, socio-economic deprivation) were generally as expected (Supplementary Tables S1 & S2). For example, admission rates for CVD and COPD increased substantially with age, and tend to be higher in more socio-economically deprived areas. In terms of other housing characteristics, dwelling type, and tenure status for LSOA housing stock were associated with admission rates. LSOAs with higher percentages of privately or socially rented properties tended to have higher admission rates. Whereas LSOAs with a higher prevalence of flats (versus houses), generally had lower admission rates; potentially due to differences in ventilation rates and thermal comfort. Rural areas had lower admission rates than urban areas, which may be a result of improved health in those areas and/or longer distances to hospitals, which typically impacts negatively on admission rates.

There were mixed findings between the air pollution measures and hospital admissions, which may be due to multi-collinearity, since the models included air pollutants strongly correlated with each other (especially PM_2.5_ and NO_2_ – see sensitivity analyses below). Notably, areas with higher minimum winter temperatures were associated with lower COPD admission rates (Risk Ratio = 0.978 [95% Confidence Interval = 0.97, 0.98] per 1 °C increase).

#### 3.1.3 Sensitivity analyses and effect modification

A range of sensitivity analyses were carried out to explore impacts of model specification (full results available on request). Restriction to winter admissions only resulted in negligible changes to rate ratios. Given the potential for multi-collinearity, models including a single deprivation indicator (income deprivation) and air pollution measure (NO_2_) were explored. These also demonstrated negligible impacts on rate ratios for energy efficiency metrics, as did categorical analysis of quintiles of mean EPC rating and investigation of modal EPC. Models stratified by urban/rural classification did not indicate differential associations.

We hypothesised that populations in areas with higher average housing energy efficiency might be more ‘resilient’ to the adverse impacts of cold winter temperatures (i.e. that the rate ratio for minimum temperature would be of greater magnitude in LSOAs with lower mean EPC). Interaction tests suggested effect modification of the minimum winter temperature association with COPD by quartile of mean EPC rating (*p* < 0.001). However, models stratified by EPC quartile produced minimum temperature rate ratios of: Quartile 1 (Q1, lowest average EPC) 0.98; Q2 0.97; Q3 0.98; Q4 0.97. There was therefore no clear pattern of stronger minimum temperature associations in lower EPC quartile areas; and in the absence of alternate explanation for the pattern observed, the significant interaction could be an artefact.

### 3.2 Devon analyses at the postcode-level

#### 3.2.1 Descriptive statistics

Despite including 500,000 households with a population of around 900,000 adults for three years, the total numbers of hospital admissions for the selected conditions were relatively low when distributed across around 35,000 postcodes across Devon. There were a total of 933 asthma, 3071 COPD, and 7905 CVD adult emergency admissions included for analyses. Around half of admissions for asthma (46%) and COPD (50%) were during the three winter months, while 34% of CVD admissions were during this colder season.

Approximately 21% of postcodes included only dwellings with loft insulation >250 mm deep; the level of insulation was mixed within 60% of postcodes, and in 15% of postcodes all dwellings had <250 mm deep loft insulation. Around 14% of postcodes included only dwellings with wall insulation, and 31% of postcodes included only dwellings with double/triple window glazing. The aggregated dwelling data indicated that the mean percentage of dwellings within a postcode with a SAP rating A-C (i.e. higher energy efficiency bands) is around 23%. In terms of tenure, 20% of households were rented from private landlords, 3.9% were rented from the local authority and 4.8% from housing associations. Weather data indicated that this area is somewhat milder, wetter, and more humid than the English average (Table 3) as would be expected given its location in the southwest of the country.

**Table 3:** Descriptive statistics for Devon postcode-level variables.

Devon Home Analytics Metrics				
Categorical measures	All %	Mixed %	None %	N/A %
Dwellings in postcode with loft insulation ≥250 mm deep	21.5	59.6	14.5	4.4^a^
Dwellings in postcode with wall insulation present	14.5	52.4	33.1	
Dwellings in postcode with full double/triple glazing	30.7	62.2	7.1	


	Mean	Std Dev	Min	Max
Continuous measures				
% Dwellings with SAP Rating A–C	23.1	34.7	0.0	100.0
Mean probability of fuel poverty	0.24	0.20	0.01	1.00

Confounders/covariates				
Indices of deprivation 2015				
Income deprivation score	0.12	0.07	0.02	0.48
Employment deprivation score	0.11	0.06	0.01	0.42
Education deprivation score	17.1	12.8	0.8	85.1

Tenure/property types				
% Households private rented	20.2	35.2	0.0	100.0
% Households local authority rented	3.9	16.0	0.0	100.0
% Households housing association rented	4.8	18.2	0.0	100.0
% Dwellings mid-terraced houses	13.8	23.0	0.0	100.0
% Dwellings semi-detached houses	28.9	31.5	0.0	100.0
% Dwellings detached houses	38.8	40.6	0.0	100.0
% Dwellings flats	18.6	31.8	0.0	100.0

Weather & air pollution				
Minimum winter temperature 2006–15 (C)	−3.0	0.5	−4.6	−1.5
Mean monthly precipitation 2006–15 (mm)	77.4	15.0	54.5	169.6
Mean relative humidity 2006–15 (%)	84.6	1.3	81.0	88.9
Mean NO2 2007–11 (ug/m^3^)	25.3	8.6	17.3	61.8
Mean ozone 2007–11 (ug/m^3^)	62.0	5.1	50.9	66.0
Mean PM_2.5_ 2007–11 (ug/m^3^)	12.5	0.5	11.3	13.3

Urban/rural classification (n)				
Urban	18,150			
Town & Fringe	6047			
Rural	11,426			

a Figures indicate the percentage of postcodes in each category; 4.4% of postcodes had no dwellings with lofts.

#### 3.2.2 Regression analyses at the postcode-level

There were no associations between loft insulation, wall insulation or glazing and asthma admissions in both the crude and adjusted models (see Supplementary Fig. S1); the relatively low numbers of admissions resulted in very wide confidence intervals around rate ratios. There was some indication that a greater extent of loft insulation was associated with higher admissions for both COPD and CVD in adjusted models. There was also a suggestion that areas with fewer wall insulated homes had lower CVD admission rates in both crude and adjusted models. There was a suggestion that all insulated dwellings in an area increased CVD hospital admissions; however, confidence intervals crossed unity in the adjusted model.

Mixed findings were also observed in the models for postcodes with more dwellings having a SAP rating of A to C and total hospital admission rates (see Supplementary Table S3). There were no associations between the increasing number of properties with SAP rating of A-C and asthma admissions. However, there is some evidence that postcodes with more dwellings SAP rated A-C had higher admission rates for COPD and CVD, although the associations were attenuated in the adjusted models.

Regression of admission rates against the probability of fuel poverty demonstrated no clear pattern of associations (see Supplementary Table S4). There was, however, an inverse association with CVD admissions, indicating that postcodes containing households with a higher mean probability of fuel poverty had lower CVD admission rates.

## 4 Discussion

This cross sectional ecological study highlighted the complexity in investigating the relationships between health and housing at the population-level using ‘big data’ approaches. While the study design prevents the ability to assess potential causal pathways, we found some evidence at the national and local level that higher hospital admission rates were found in areas where average household energy efficiency levels were greater. For example, there was some evidence of around a 0.5 to 1.0% increase in hospital admission rates for asthma, COPD, and CVD. A similar trend was observed in the Devon analyses where higher average SAP rating (i.e. higher % properties rated A-C) at the postcode-level were associated with increased admission rates for asthma, COPD and CVD. However, these associations were attenuated in the adjusted model due to a relatively small number of hospital admissions.

### 4.1 Synthesis with existing knowledge

The relationship between the built environment and human health is complex and possibly contributes to our mixed findings. Resultant health outcomes are modified by complex interactions between diverse direct and indirect factors such as a range of individual behaviours, socio-cultural factors, and changes in the built and natural environments (Sharpe et al., 2018b). The potential health effects of these household interventions, changes in ambient air temperature, fuel poverty/resident behaviours, indoor air quality, and the variability of energy efficiency measures are discussed further in the following sections.

#### 4.1.1 Health benefits of household energy efficiency interventions

Living in fuel poverty is a distinct societal challenge, which poses a significant risk to the physical and mental health of those living in cold homes (Liddell and Morris, 2010). For this reason, fuel poverty policies and the promotion of energy efficiency improvements must be supported to deliver the co-benefits of meeting carbon reduction and fuel poverty alleviation targets. Our study further supports the need to shift away from prior energy efficiency policies that have led to the implementation of interventions that fail to account for whole house approaches (discussed below). This includes those that account for changes in resident behaviours/lifestyles and the entire property, which includes the consideration of improved insulation, heating and ventilation (i.e. more holistic approaches) (Sharpe et al., 2018b). Evidence from the US supports the uptake of more sustainable green building programmes that can reduce energy use/cost, carbon emissions, reduce hospital admissions, and lost work and school days; pursuing these approaches provides an important and previously unquantified societal value (MacNaughton et al., 2018). For individual households, energy efficiency measures (with external wall insulation being the most effective) make homes more affordable to heat and thus improve the thermal comfort of homes (i.e. achieving the recommended indoor temperature of 18-24 °C) and reduce daily gas use by up to 37% (Poortinga et al., 2017).

It is generally accepted that home improvements in terms of draughtt proofing and making homes more affordable to heat can reduce cold-related winter illnesses. While there are some mixed findings, household energy efficiency improvements can on average have a small but positive effect on improving resident health. The greatest health gains may be delivered by targeting more vulnerable populations susceptible to cold homes (Maidment et al., 2014; Thomson et al., 2013). In a previous health impact modelling study, household energy efficiency interventions improved health through reduced exposure to cold and indoor air pollutants if delivered alongside improved ventilation (Hamilton et al., 2015). Drawing from studies from New Zealand, well-designed randomised controlled trials involving a range of energy efficiency measures have shown consistent improvements in resident health. Although this was not consistent across all of the health outcomes measured (Howden-Chapman et al., 2011; Howden-Chapman et al., 2007; Howden-Chapman et al., 2008); and it is not clear how generalizable these findings are to UK housing stock.

Improvements including improved insulation and heating upgrades in fuel poor homes across Wales have been found to reduce respiratory related general practitioner visits and hospital admissions relating to respiratory and cardiovascular events, although the effect did not reach the level of statistical significance due to the small number of admissions (Welsh Government, 2017). Due to the study design, this Welsh study did not account for long-term changes in health status, which is an important factor to consider. Similarly, another retrospective study in Carmarthenshire, UK also found that hospital admissions could be avoided through home improvements, which included interventions such as upgrading electrical systems, and improved windows, doors and insulation. However, upgrading heating systems, loft insulation, kitchens, and bathrooms had no effect on hospital admissions (Rodgers et al., 2018). Another study found no associations between improving the energy performance of homes and emergency hospital admissions (Poortinga et al., 2018).

This demonstrates that different interventions can influence the health outcomes of interest, which is evident in our study and others (Maidment et al., 2014). However, it is important to consider the potential impact of different study designs, exposure and case definitions and treatment of diverse confounders. While there are overwhelming benefits resulting from well-designed energy efficiency interventions, there is the potential for a range of modifiable risk factors and unintended consequences (Shrubsole et al., 2014). These can result from changes in ambient air temperature, the severity of fuel poverty, impacts on indoor air quality and health, and variability of energy efficiency measures described below.

#### 4.1.2 Changes in ambient air temperature

A further explanation to our findings may be due to differences in minimum temperatures and energy efficiency levels. We found that areas with higher minimum winter temperatures tended to have lower COPD admissions. Minimum ambient air temperatures have been previously found to increase of cardio-respiratory outcomes (Bai et al., 2017; Cong et al., 2017; Phung et al., 2016), which vary by locality and country (Guo et al., 2014; Guo et al., 2016). However, we found no association between lower minimum temperatures with either asthma or CVD admission rates. Furthermore, the relationship between minimum winter temperatures, energy efficiency levels and admission rates did not change in our effect modification analyses. Despite these findings, it is thought that fuel poverty interventions will play a key role in reducing cold-related mortality and morbidity (Hajat, 2017).

#### 4.1.3 Fuel poverty and resident behaviours

Equally, it is also possible that some interventions can have a detrimental effect on health (Maidment et al., 2014; Sharpe et al., 2015d) in some populations. The resultant impact of health may be a result of overall poverty and low social economic status, which is compounded by an inability to adequately heat and ventilate the home. Due to the cost of living (Committee on Climate Change, 2017), energy efficiency improvements may not eliminate the risk of cold on the lowest income households (Anderson et al., 2012) nor take full account of resident behaviours, risk perception and choices when heating and ventilating the home (Critchley et al., 2007; Sharpe et al., 2015c). Therefore, the potential benefits of fuel poverty alleviation programmes could be overshadowed by rising energy prices (Howden-Chapman et al., 2012). Consequently, some households may continue to ration their heating, despite home improvements (Lomax and Wedderburn, 2009).

This means that some home improvements may not help the most fuel poor avoid the potential impact of living in cold and damp homes. Homes receiving energy efficiency interventions may continue to experience problems with mould contamination (Richardson et al., 2005), regardless of occupant risk perception of the potential health impacts, heating and ventilation practices and energy efficiency levels (Sharpe et al., 2015c). Resultant cold homes and associated indoor air pollutants such as mould contamination (and associated air pollutants) can lead to a range of adverse health effects (Fisk et al., 2010; Fisk et al., 2007; Quansah et al., 2012; Sharpe et al., 2015a).

#### 4.1.4 Impacts on indoor air quality and health

Despite the good intentions of the Government and activities of responsible companies, there are concerns over the poor quality of household energy efficiency installations (Bonfield, 2016). Poorly designed and implemented energy efficiency upgrades means that household ventilation rates may be significantly reduced, which can be further compounded by resident behaviours (as discussed above). Despite some inconsistent evidence (Frey et al., 2015), this can lead to reduced air quality (Howieson, 2014), and result in, for example, increased exposures to indoor pollutants such as radon (Milner et al., 2014; Symonds, 2019), carbon monoxide (Pigg et al., 2017), nitrogen dioxide/nitrogen oxides and formaldehyde (Coombs et al., 2016). The impact on air quality can lead to poorer physical and psychosocial health outcomes (Grey et al., 2017a; Milner et al., 2014; Sharpe et al., 2015d). This is important to consider because the majority of age groups spend between 70%–80% or more of their time indoors (Lavergea et al., 2011), which increases in the very young and the elderly (Briggs et al., 2003).

Resultant health outcomes are likely to be dependent on occupant lifestyles, changes in ventilation patterns (i.e. resident behaviours), the type of building, and interactions between indoor and outdoor sources of air pollutants (Coombs et al., 2016; Das et al., 2013; Taylor et al., 2016), Other factors include different disease phenotypes or endotypes (Lötvall et al., 2011) and/or the ability of patients to self-manage these chronic diseases (Morell et al., 2014; Mosnaim et al., 2016). Another important factor that could further explain our findings is the variability and/or quality of building/energy improvement measures installed across England.

#### 4.1.5 Variability of energy efficiency measures

Prior fuel poverty policies have targeted more vulnerable populations such as low income households or those vulnerable to cold, which may explain an increase in hospital admission rates in areas of greater energy efficiency levels. In terms of variability in measures, these are likely to differ in terms of the quality and maintenance of homes improvements, as well as the consistency of measures being installed. Building on prior studies (Richardson et al., 2005; Sharpe et al., 2015d; Shrubsole et al., 2014), our findings contribute to the increasing literature in this area suggesting negative outcomes, which may be due to a lack of ‘whole house’ approaches (i.e. interventions that consider the performance of the whole house and resident behaviours) (Sharpe et al., 2018b). For example, installing single energy efficiency measures such as glazing or insulation or heating alone may not improve the indoor environment and subsequent health outcomes, observed by Maidment et al. (2014) and Rodgers et al. (2018).

Other factors to consider are the impact of tenure and motivations for improving and maintaining home energy efficiency measures. While those living in social housing generally experience higher quality housing when compared to other tenures (e.g. due to decent homes standard) (Sharpe et al., 2015d), the uptake of measures within the home owner sector depends on diverse economic, social and environmental motivations (Organ et al., 2013). Furthermore, our population-level approach may also conceal the benefits experienced by more vulnerable populations such as those with a chronic respiratory disease (Thomson et al., 2009; Thomson et al., 2013) and/or those living in older buildings (i.e. those in most need) (Howden-Chapman et al., 2011) for example. Further investigations are also needed to explore how changes in household energy efficiency influence and modify indoor air quality and how this in turn impacts the health of residents. This should include an assessment of other potential sources of indoor air pollutants (Royal Colleage of Physicians, 2016) that could further modify indoor air quality and resultant health outcomes. Including an improved understanding into the complex and overlapping risks associated with diverse biological allergens (Sharpe et al., 2015e), other physical (particulates) and chemical (e.g. nitrogen dioxide) agents. These need to be put into context with variable resident behaviours, heating and ventilation patterns, which modify exposure to these indoor air pollutants (Sharpe et al., 2015b).

### 4.2 Limitations

This population-level study enabled us to explore the relationship between changes in household energy efficiency levels and health. We utilised prior energy efficiency and health metrics such as SAP/EPC ratings and HES (respectively), which have been previously used (Donaldson and Wedzicha, 2006; Sharpe et al., 2015d). However, there has been some confusion with the use and quality of energy efficiency metrics such as EPCs in building sector (Jenkins et al., 2017; Pérez-Lombard et al., 2009). Also, these are unable to account for day-to-day changes in energy performance, which is modified by resident behaviours (Sharpe et al., 2015d). We accounted for hospital admission diagnosis codes which appeared in the first or second diagnosis field, which could potentially result in an underestimate of admissions where the condition of interest appears in a subsequent diagnosis field. However, this is unlikely to be a significant source of error, and by focussing on asthma, COPD and CVD as primary reasons for hospitalisation means that we avoid including admissions where these conditions are comorbidities or sequelae.

Further limitations include the study's ecological study design and the ‘ecological fallacy’ (Savitz, 2012), which assumes that exposures assigned on the population-level apply to an individual. There is also the potential for chance findings due to the large sample sizes and number of statistical analyses conducted to explore our hypotheses. The cross-sectional nature of the study means that we cannot assess the temporal sequence of exposures (home energy efficiency changes) and health outcomes.

As described above, our findings may have been influenced by reverse causality (i.e. those vulnerable to cold and ill may be more likely to receive fuel poverty interventions). While accounting for ambient environmental conditions, these were based on modelled data sets, which can also include an element of error. For example air pollution data were based on well-validated models, but even still at 1x1km resolution will miss the fine spatial variability of pollution concentrations (e.g. those close to busy roads); in this case exposure estimation error is likely to be greater for high resolution local analyses relative to lower resolution national analyses.

As is common with this type of observational study, especially in the local analyses that depend partly on modelled estimates of housing energy efficiency, it is likely that there is a degree of misclassification of the exposures of interest. This may have led to bias in the measured associations between exposures and outcomes, most likely toward the null if misclassification is non-differential. Potential misclassification arises through both exposure estimation error (e.g. since efficiency metrics for the local study are partially based on modelled values) and through inconsistency in the timescales of data. The exposure variables used varied from those used in other data sets such as the English Housing Survey (Hamilton et al., 2014), which could result from differences in definitions used, type of sampling stratification, collection/recording methodologies, and missing records (Hamilton et al., 2014).

Using aggregated data sets makes it difficult to control for individual confounding factors such as those associated with smoking status, weight, and or ethnicity, which are known to influence cardio-respiratory outcomes. Due to the nature of the housing data sets, we were unable to account for other potential confounders such as the interaction between emissions from cooking and heating appliances and/or the interaction with variable heating and ventilation patterns. We were also unable to fully account for the proximity of the home to the hospital, which can influence admission rates. Also, the HA modelled the energy efficiency measures across around 50% of the housing stock across Devon, which may contribute to some of the mixed findings.

### 4.3 Study implication

As indicated above, our study builds on existing evidence in the support of delivering more sustainable housing interventions that take a more ‘whole house’ approach are urgently needed to avoid the potential short-term benefits and unintended consequences of some energy efficiency programmes (Sharpe et al., 2015b). Future research needs to take a more holistic approach to delivering healthier indoor environments, which consider a dynamic and complex system with multiple interactions between a range of factors. These need to consider: the bio-psycho-social aspects of health; the complex interaction between resident behaviours and the built environment; the impacts of climate change; and the adopted energy efficiency measures and indoor environments (Vardoulakis et al., 2015; Wierzbicka et al., 2018). Future interventions should draw from international best practices that consider changes in the built environment and resident behaviours.

Some positive examples include the ‘Green public housing’ or ‘Healthy Homes’ initiatives (Breysse et al., 2011; Colton et al., 2015), but these need to be put into context with the built environment and resident behaviours in the UK. Those that consider the behavioural and physical environments have led to demonstrated improved overall health, asthma, and non-asthma respiratory symptoms (Breysse et al., 2011).

Therefore, well-designed salutogenic improvements to household energy efficiency that consider resident behaviours and greater ventilation rates (with heat recovery and heating systems) such as those in the US ASHRAE standard (Francisco et al., 2017) have the potential to improve long-term health outcomes. Importantly, these must account for residents ability and willingness to pay for heating and be delivered alongside resident training (Sharpe et al., 2015d), community engagement (Grey et al., 2017b) and improved awareness among housing and health practitioners (Mc Conalogue et al., 2016) to achieve more sustainable housing interventions. Furthermore, the development of improved area-based targeting tools may help to identify and target those most in need of energy efficiency improvements (Walker et al., 2014).

## 5 Conclusion

In this population-level study, we found some evidence that higher average area-level energy efficiency ratings were associated with a small but statistically significant increase in hospital admission rates. While this is in contrast to our original hypothesis, it is important to consider that some of the findings showed attenuated effects after adjustment. Furthermore, our analyses across the local analyses do not necessarily support this conclusion, highlighting the complexity in modelling investigating links between changes in the built environment and human health. There are some suggestions in our findings of positive impacts on health outcomes of boiler replacements, which have the potential to improve energy efficiency and home warmth without ventilation penalty. The inconsistency these findings and limitations of the ecological design further supports the need for more complex modelling and/or larger natural experiments at the household and individual-level. This requires improved data sharing and information governance of large population-level data sets; improved fiscal incentives (e.g. flexible eligibility criteria); and the targeting of more vulnerable populations who may benefit most from home improvements. Despite the limitations of this study, it is consistent with evidence indicating a need for energy efficiency measures to be put in place in a truly sustainable way. A more holistic and ‘whole house’ approach needs to be taken that considers the physical built environment, communities, variable cultures, lifestyles, and resident behaviours.

## Funding

This study was supported and funded by the Eaga Charitable Trust, with additional support from the University of Exeter Medical School and the Public Health team in Cornwall Council. The research was also supported in part by the National Institute for Health Research Health Protection Research Unit (NIHR HPRU) in Environmental Change and Health at the London School of Hygiene and Tropical Medicine in partnership with Public Health England, and in collaboration with the University of Exeter, University College London, and the Met Office (HPRU-2012-10016). Weather and air pollution data were provided via the MEDMI project: U.K. Medical Research Council (MRC) and the U.K. Natural Environment Council (NERC) for the MEDMI Project (MR/K019341/1). Additional funding was provided by the South West Academic Health Science Network [grant number SW AHSN G005] and the European Regional Development Fund [grant number SZ07660] for the SMARTLINE Project and the European Commission Horizon 2020 funded INHERIT project, coordinated by EuroHealthNet [grant number 667364]. Jonathon Taylor JT is funded by the Wellcome Trust ‘Our Planet, Our Health’ award Complex Urban Systems for Sustainability and Health (209387/Z/17/Z).

## Declaration of competing interest

The authors declare the following financial interests/personal relationships which may be considered as potential competing interests: IH is a consultant who has worked with Energy Saving Trust (EST). Both IH and EST were involved in the development and implementation of this project. EST is an organisation providing consultancy and consumer information, including programmes for governments on energy efficiency policy.

## Appendix A Supplementary data

Supplementary material Image 1
