# Supplementary material for: Household energy efficiency and health: Area-level analysis of hospital admissions in England
Source: Environ Int. 2019 Dec;133(Pt A):105164. doi: 10.1016/j.envint.2019.105164 (PMC6853278; doi:10.1016/j.envint.2019.105164)
Supplement: Supplementary file 1 — Supplementary material [file mmc1.docx]

## Supplementary Material

## Full regression model results

**Table S1. National: Hospital admissions (3-year totals) and energy efficiency measures: full model results (n=31,481 LSOAs)**

|  | **Total asthma admissions** | | | **Total COPD admissions** | | | **Total CVD admissions** | | |
| --- | --- | --- | --- | --- | --- | --- | --- | --- | --- |
|  | **RR** | **95% CI** | **p** | **RR** | **95% CI** | **p** | **RR** | **95% CI** | **p** |
| Loft insulation ^a^ | 1.004 | [1.004,1.005] | <0.001 | 1.002 | [1.001,1.003] | <0.001 | 1.004 | [1.003,1.004] | <0.001 |
| Wall insulation ^b^ | 1.000 | [0.998,1.002] | 0.798 | 1.002 | [0.999,1.004] | 0.155 | 0.999 | [0.998,1.001] | 0.356 |
| Full double/triple glazing ^c^ | 0.999 | [0.997,1.000] | 0.128 | 0.999 | [0.998,1.001] | 0.476 | 0.999 | [0.998,1.000] | 0.011 |
| Rate of draught proofing measures ^d^ | 1.000 | [0.999,1.001] | 0.814 | 1.002 | [1.002,1.003] | <0.001 | 1.000 | [1.000,1.001] | 0.282 |
| Rate of boiler replacement measures ^e^ | 0.998 | [0.994,1.002] | 0.398 | 0.992 | [0.987,0.996] | 0.001 | 1.000 | [0.996,1.004] | 0.935 |
| Age group 18-29 | 1.505 | [1.477,1.534] | <0.001 | 0.005 | [0.004,0.005] | <0.001 | 0.012 | [0.011,0.012] | <0.001 |
| 30-44 | 1.284 | [1.261,1.307] | <0.001 | 0.028 | [0.027,0.030] | <0.001 | 0.089 | [0.087,0.090] | <0.001 |
| 45-59 | 1.191 | [1.171,1.212] | <0.001 | 0.244 | [0.240,0.249] | <0.001 | 0.471 | [0.467,0.476] | <0.001 |
| 60-69 | 1.000 | Ref |  | 1.000 | Ref |  | 1.000 | Ref |  |
| 70-79 | 1.367 | [1.338,1.396] | <0.001 | 2.183 | [2.152,2.214] | <0.001 | 2.056 | [2.038,2.073] | <0.001 |
| 80-89 | 1.826 | [1.786,1.867] | <0.001 | 3.452 | [3.400,3.504] | <0.001 | 4.015 | [3.978,4.051] | <0.001 |
| 90+ | 2.202 | [2.126,2.282] | <0.001 | 3.489 | [3.408,3.571] | <0.001 | 6.430 | [6.350,6.512] | <0.001 |
| Male | 1.000 | Ref |  | 1.000 | Ref |  | 1.000 | Ref |  |
| Female | 1.874 | [1.853,1.894] | <0.001 | 0.776 | [0.768,0.785] | <0.001 | 0.605 | [0.601,0.608] | <0.001 |
| Income deprivation score | 4.716 | [3.964,5.612] | <0.001 | 0.733 | [0.609,0.883] | 0.001 | 3.536 | [3.157,3.961] | <0.001 |
| Employment deprivation score | 1.321 | [1.048,1.666] | 0.019 | 14.595 | [11.437,18.625] | <0.001 | 1.514 | [1.296,1.768] | <0.001 |
| Education deprivation score | 1.003 | [1.002,1.004] | <0.001 | 1.009 | [1.008,1.010] | <0.001 | 1.003 | [1.002,1.003] | <0.001 |
| % properties private rented | 1.004 | [1.003,1.005] | <0.001 | 1.013 | [1.012,1.014] | <0.001 | 1.005 | [1.004,1.005] | <0.001 |
| % properties social rented | 1.003 | [1.002,1.003] | <0.001 | 1.014 | [1.013,1.015] | <0.001 | 1.001 | [1.001,1.002] | <0.001 |
| % flats (of all dwellings) | 0.997 | [0.997,0.998] | <0.001 | 0.997 | [0.996,0.998] | <0.001 | 0.998 | [0.997,0.998] | <0.001 |
| Urban | 1.000 | Ref |  | 1.000 | Ref |  | 1.000 | Ref |  |
| Town & Fringe | 0.954 | [0.934,0.974] | <0.001 | 0.968 | [0.946,0.990] | 0.006 | 0.994 | [0.981,1.007] | 0.342 |
| Rural | 0.896 | [0.873,0.919] | <0.001 | 0.848 | [0.825,0.871] | <0.001 | 0.936 | [0.923,0.950] | <0.001 |
| Minimum winter temp C | 1.005 | [0.998,1.013] | 0.164 | 0.977 | [0.969,0.984] | <0.001 | 1.000 | [0.995,1.004] | 0.899 |
| Mean monthly precipitation mm | 1.003 | [1.002,1.003] | <0.001 | 1.001 | [1.001,1.002] | <0.001 | 1.002 | [1.002,1.003] | <0.001 |
| Mean relative humidity % | 0.989 | [0.984,0.994] | <0.001 | 1.006 | [1.001,1.011] | 0.026 | 1.000 | [0.997,1.003] | 0.95 |
| Mean NO2 (ug/m3) | 1.002 | [1.000,1.004] | 0.104 | 1.001 | [0.999,1.003] | 0.35 | 0.999 | [0.998,1.000] | 0.228 |
| Mean O3 (ug/m3) | 1.004 | [1.001,1.007] | 0.003 | 0.992 | [0.989,0.995] | <0.001 | 0.999 | [0.998,1.001] | 0.367 |
| Mean PM2.5 (ug/m3) | 0.946 | [0.928,0.965] | <0.001 | 0.967 | [0.949,0.987] | 0.001 | 0.940 | [0.930,0.951] | <0.001 |

a. ≥250mm deep loft insulation per 100 dwellings; b. Wall insulation per 100 dwellings; c. Full double/triple glazing per 100 dwellings; d. Measures per 100 dwellings; e. Measures per 100 dwellings.

RR: Rate Ratio; CI: Confidence Interval; p: p-value

**Table S2. National: Hospital admissions (3-year totals) and mean LSOA Energy Performance Certificate Rating: full model results (n=31,481 LSOAs)**

|  | **Total asthma admissions** | | | **Total COPD admissions** | | | **Total CVD admissions** | | |
| --- | --- | --- | --- | --- | --- | --- | --- | --- | --- |
|  | **RR** | **95% CI** | **p** | **RR** | **95% CI** | **p** | **RR** | **95% CI** | **p** |
| LSOA mean EPC Rating | 1.005 | [1.004,1.006] | <0.001 | 1.011 | [1.010,1.013] | <0.001 | 1.006 | [1.005,1.007] | <0.001 |
| Age group 18-29 | 1.500 | [1.471,1.529] | <0.001 | 0.005 | [0.004,0.005] | <0.001 | 0.012 | [0.011,0.012] | <0.001 |
| 30-44 | 1.280 | [1.257,1.303] | <0.001 | 0.028 | [0.027,0.029] | <0.001 | 0.088 | [0.087,0.090] | <0.001 |
| 45-59 | 1.190 | [1.169,1.211] | <0.001 | 0.244 | [0.240,0.249] | <0.001 | 0.471 | [0.466,0.475] | <0.001 |
| 60-69 | 1.000 | Ref |  | 1.000 | Ref |  | 1.000 | Ref |  |
| 70-79 | 1.368 | [1.339,1.397] | <0.001 | 2.183 | [2.153,2.214] | <0.001 | 2.056 | [2.039,2.074] | <0.001 |
| 80-89 | 1.828 | [1.788,1.869] | <0.001 | 3.451 | [3.400,3.504] | <0.001 | 4.017 | [3.981,4.054] | <0.001 |
| 90+ | 2.205 | [2.128,2.284] | <0.001 | 3.491 | [3.411,3.574] | <0.001 | 6.438 | [6.357,6.520] | <0.001 |
| Male | 1.000 | Ref |  | 1.000 | Ref |  | 1.000 | Ref |  |
| Female | 1.874 | [1.853,1.894] | <0.001 | 0.775 | [0.767,0.784] | <0.001 | 0.604 | [0.601,0.608] | <0.001 |
| Income deprivation score | 5.356 | [4.478,6.406] | <0.001 | 0.802 | [0.668,0.963] | 0.018 | 4.061 | [3.621,4.554] | <0.001 |
| Employment deprivation score | 1.521 | [1.206,1.918] | <0.001 | 17.353 | [13.597,22.148] | <0.001 | 1.736 | [1.484,2.032] | <0.001 |
| Education deprivation score | 1.003 | [1.002,1.004] | <0.001 | 1.009 | [1.008,1.009] | <0.001 | 1.003 | [1.002,1.003] | <0.001 |
| % properties private rented | 1.003 | [1.002,1.004] | <0.001 | 1.013 | [1.012,1.014] | <0.001 | 1.004 | [1.004,1.005] | <0.001 |
| % properties social rented | 1.001 | [1.000,1.002] | 0.003 | 1.013 | [1.012,1.014] | <0.001 | 1.000 | [0.999,1.001] | 0.880 |
| % flats (of all dwellings) | 0.997 | [0.996,0.997] | <0.001 | 0.996 | [0.995,0.997] | <0.001 | 0.997 | [0.997,0.998] | <0.001 |
| Urban | 1.000 | Ref |  | 1.000 | Ref |  | 1.000 | Ref |  |
| Town & Fringe | 0.952 | [0.932,0.973] | <0.001 | 0.969 | [0.947,0.992] | 0.008 | 0.994 | [0.981,1.007] | 0.369 |
| Rural | 0.902 | [0.878,0.927] | <0.001 | 0.881 | [0.856,0.906] | <0.001 | 0.954 | [0.939,0.968] | <0.001 |
| Minimum winter temp C | 1.003 | [0.996,1.011] | 0.386 | 0.978 | [0.970,0.986] | <0.001 | 0.998 | [0.994,1.002] | 0.372 |
| Mean monthly precipitation mm | 1.003 | [1.002,1.003] | <0.001 | 1.002 | [1.001,1.002] | <0.001 | 1.002 | [1.002,1.003] | <0.001 |
| Mean relative humidity % | 0.994 | [0.989,0.999] | 0.029 | 1.011 | [1.005,1.016] | <0.001 | 1.005 | [1.002,1.008] | 0.001 |
| Mean NO2 (ug/m3) | 1.001 | [1.000,1.003] | 0.142 | 1.001 | [0.999,1.003] | 0.523 | 0.999 | [0.998,1.000] | 0.096 |
| Mean O3 (ug/m3) | 1.003 | [1.001,1.006] | 0.012 | 0.990 | [0.987,0.993] | <0.001 | 0.998 | [0.997,1.000] | 0.044 |
| Mean PM2.5 (ug/m3) | 0.928 | [0.911,0.946] | <0.001 | 0.957 | [0.939,0.976] | <0.001 | 0.926 | [0.916,0.936] | <0.001 |

**Table S3. Local: Hospital admissions and SAP Rating model results (n=35,623 postcodes)**

| **Total 3 year admissions** | **Crude model** | | | **Fully adjusted model** | | |
| --- | --- | --- | --- | --- | --- | --- |
| **Postcode % SAP ABC** | **RR** | **95% CI** | **p** | **RR** | **95% CI** | **p** |
| **Asthma** |  |  |  |  |  |  |
| 0-<0.25 | 1.000 | Ref |  | 1.000 | Ref |  |
| 0.25-<0.50 | 1.396 | [0.91, 2.13] | 0.123 | 1.279 | [0.85, 1.91] | 0.232 |
| 0.50-<0.75 | 1.157 | [0.82, 1.64] | 0.413 | 1.033 | [0.74, 1.44] | 0.847 |
| 0.75-1.00 | 1.207 | [0.92, 1.58] | 0.172 | 0.896 | [0.67, 1.19] | 0.453 |
| **COPD** |  |  |  |  |  |  |
| 0-<0.25 | 1.000 | Ref |  | 1.000 | Ref |  |
| 0.25-<0.50 | *1.596* | *[1.21, 2.11]* | *0.001* | 1.137 | [0.87, 1.49] | 0.348 |
| 0.50-<0.75 | *1.541* | *[1.22, 1.94]* | *<0.001* | 0.939 | [0.74, 1.19] | 0.608 |
| 0.75-1.00 | *2.161* | *[1.83, 2.55]* | *<0.001* | 0.878 | [0.73, 1.06] | 0.175 |
| **CVD** |  |  |  |  |  |  |
| 0-<0.25 | 1.000 | Ref |  | 1.000 | Ref |  |
| 0.25-<0.50 | 0.964 | [0.85, 1.09] | 0.567 | 0.892 | [0.79, 1.01] | 0.063 |
| 0.50-<0.75 | *1.163* | *[1.04, 1.30]* | *0.009* | 1.046 | [0.93, 1.17] | 0.438 |
| 0.75-1.00 | *1.356* | *[1.25, 1.47]* | *<0.001* | 1.053 | [0.97, 1.15] | 0.244 |

**Table S4. Local: Hospital admissions and probability of fuel poverty model results (n=35,623 postcodes)**

| **Total 3 year admissions** | **Crude model** | | | **Fully adjusted model** | | |
| --- | --- | --- | --- | --- | --- | --- |
| **Postcode mean fuel poverty probability** | **RR** | **95% CI** | **p** | **RR** | **95% CI** | **p** |
| **Asthma** |  |  |  |  |  |  |
| 0-<0.25 | 1.000 | Ref |  | 1.000 | Ref |  |
| 0.25-<0.50 | 1.141 | [0.68, 1.91] | 0.616 | 1.056 | [0.72, 1.55] | 0.780 |
| 0.50-<0.75 | 0.793 | [0.47, 1.34] | 0.386 | 0.701 | [0.38, 1.28] | 0.246 |
| 0.75-1.00 | 0.753 | [0.26, 2.18] | 0.601 | 0.635 | [0.22, 1.81] | 0.395 |
| **COPD** |  |  |  |  |  |  |
| 0-<0.25 | 1.000 | Ref |  | 1.000 | Ref |  |
| 0.25-<0.50 | *0.684* | *[0.55, 0.85]* | *0.001* | 0.938 | [0.75, 1.18] | 0.583 |
| 0.50-<0.75 | *0.610* | *[0.42, 0.88]* | *0.009* | 0.958 | [0.65, 1.42] | 0.831 |
| 0.75-1.00 | 0.756 | [0.42, 1.35] | 0.347 | 1.255 | [0.71, 2.23] | 0.438 |
| **CVD** |  |  |  |  |  |  |
| 0-<0.25 | 1.000 | Ref |  | 1.000 | Ref |  |
| 0.25-<0.50 | 0.940 | [0.87, 1.02] | 0.141 | 0.919 | [0.84, 1.01] | 0.085 |
| 0.50-<0.75 | *0.835* | *[0.72, 0.96]* | *0.014* | *0.771* | *[0.66, 0.90]* | *0.001* |
| 0.75-1.00 | 0.800 | [0.61, 1.05] | 0.102 | *0.714* | *[0.54, 0.95]* | *0.019* |

**Figure S1 energy efficiency metric associations with 3-year total hospital admission rates (Devon)**

**
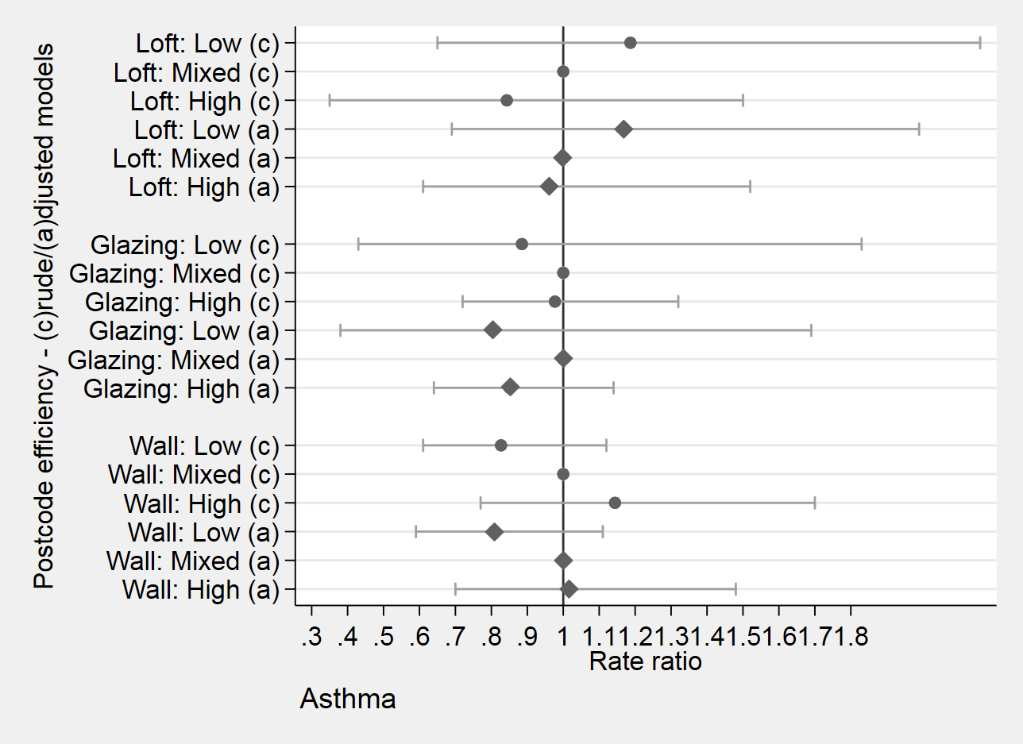
**

**
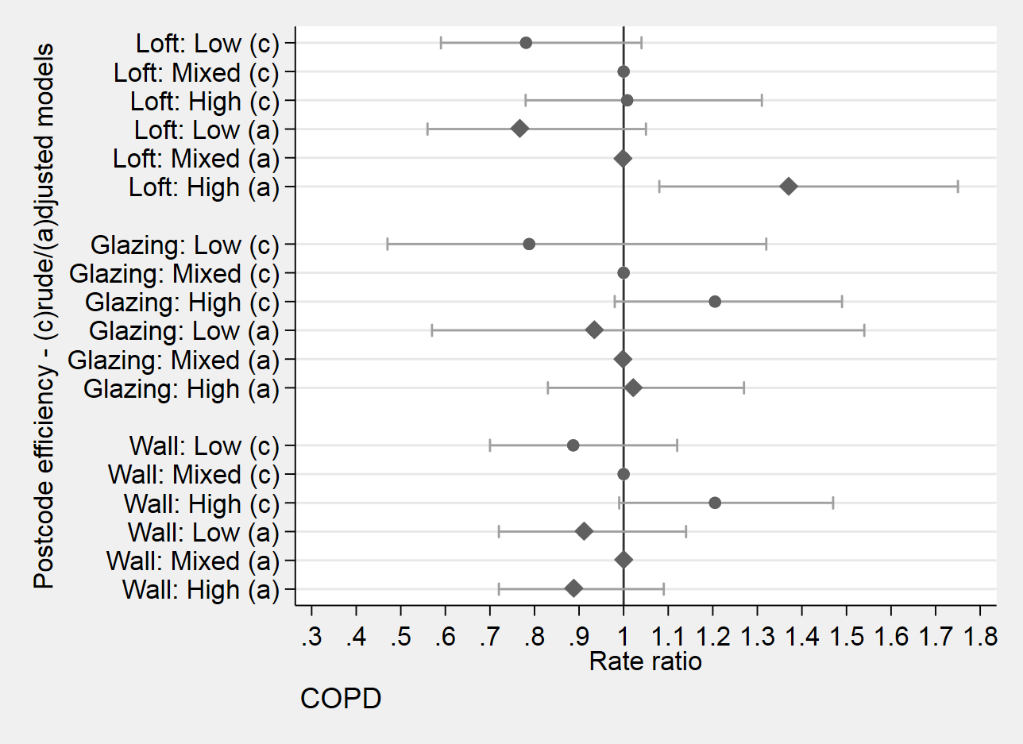
**

**
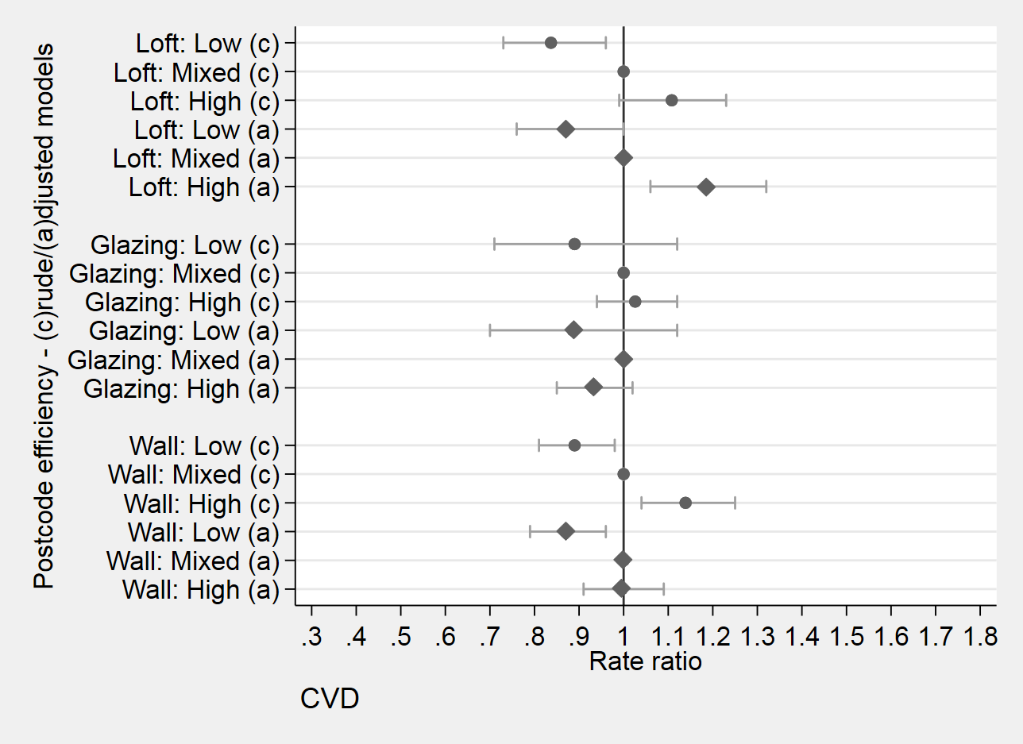
**

Low: None of the dwellings in the postcode have the efficiency measure in place; High: All of the dwellings in the postcode have the efficiency measure in place; Mixed: A mixture of dwellings with/without the efficiency measure within the postcode.
